# Development of a New Electrochemical Sensor Based on Mag-MIP Selective Toward Amoxicillin in Different Samples

**DOI:** 10.3389/fchem.2021.615602

**Published:** 2021-03-19

**Authors:** Rosario López, Sabir Khan, Ademar Wong, María del Pilar Taboada Sotomayor, Gino Picasso

**Affiliations:** ^1^Laboratory of Physical Chemistry Research, Faculty of Sciences, National University of Engineering, Lima, Peru; ^2^Analytical Department, Chemistry Institute, State University of São Paulo (UNESP), Araraquara, Brazil; ^3^National Institute for Alternative Technologies of Detection, Toxicological Evaluation & Removal of Micropollutants and Radioactives (INCT-DATREM), Araraquara, Brazil

**Keywords:** mag-MIP, amoxicillin, electrochemical sensor, selective adsorption, carbon paste electrode

## Abstract

This work describes an electrochemical sensor for the selective recognition and quantification of amoxicillin and a β-lactam antibiotic in real samples. This sensor consists of a carbon paste electrode (CPE) modified with mag-MIP (magnetic molecularly imprinted polymer), which was prepared by precipitation method via free radical using acrylamide (AAm) as functional monomer, N,N′-methylene*bis*acrylamide (MBAA) as a crosslinker, and potassium persulfate (KPS) as initiator, to functionalized magnetic nanoparticles. The magnetic non-imprinted polymers (mag-NIP) were prepared using the same experimental procedure without analyte and used for the preparation of a CPE for comparative studies. The morphological, structural, and electrochemical characteristics of the nanostructured material were evaluated using Field emission gun scanning electron microscopy (FEG-SEM), Transmission electron microscopy (TEM), Fourier transform infrared spectroscopy (FTIR), Vibrating sample magnetometry (VSM), X-ray diffraction (XRD), and voltammetric technique. Electrochemical experiments performed by square wave voltammetry show that the mag-MIP/CPE sensor had a better signal response compared to the non-imprinted polymer-modified electrode (mag-NIP/CPE). The sensor showed a linear range from 2.5 to 57 μmol L^−1^ of amoxicillin (*r*^2^ = 0.9964), with a limit of detection and a limit of quantification of 0.75 and 2.48 μmol L^−1^, respectively. No significant interference in the electrochemical signal of amoxicillin was observed during the testing experiments in real samples (skimmed milk and river water). The proposed mag-MIP/CPE sensor could be used as a good alternative method to confront other techniques to determine amoxicillin in different samples.

## Introduction

Pharmaceuticals are used domestically in veterinary medicine and hospitals, and could be analgesics, antibiotics, anti-inflammatory, antiepileptic, antidepressants, hormones, statins, beta-blockers, and products of contrasts, among others (Miège et al., 2009). Drugs that have been administrated to a large extent in humans or animals are largely excreted directly through the urine (unmetabolized 55–80%) and feces and other parts of the body (Verlicchi et al., 2010) that persist in the environment. Among pharmaceuticals, antibiotics are the most widely used in the treatment of bacterial infections, for improving health in humans, for preventing and treating animals and plant infections, and promoting growth in animal farming (Martinez, 2009).

Antibiotics can reach the environment by various means, such as excretion in urine and feces, direct disposal of unused or expired drugs by drug factories, hospitals, and veterinary surgeries (Christou et al., 2017). The presence of antibiotics in the environment, for example in natural water and soil, principally causes antibiotic resistance, hypersensitivity reaction, and aplastic anemia in sensitive humans. Antibiotic resistance is an adaptative genetic natural phenomenon possessed or acquired by bacteria in which it can grow and survive, even when in the presence of an antibiotic of therapeutic concentration. In this way, multiple-antibiotic-resistant pathogenic bacteria exist in the environment, known as superbugs (Carvalho and Santos, 2016). The World Health Organization (WHO) outlines that at least 700,000 people die each year due to drug-resistant diseases. Of these, ~230,000 people die from multidrug-resistant tuberculosis, as well as common diseases, including respiratory tract infections, sexually transmitted infections, and urinary tract infections, which are untreatable (World Health Organization, 2019).

Amoxicillin is a β-lactam antibiotic semi-synthetic widely used in human and veterinary medicine for the treatment of infections since it has a wide spectrum of action in gram-positive and gram-negative bacteria. For this reason, amoxicillin is used as the first-line antibiotic in different countries around the world, including Italy, the UK, Australia, Brazil, and Korea, which have the highest sales (World Health Organization, 2019). Globally, amoxicillin is one of the most used and prescribed antibiotics, especially in the public sector for the treatment of colds, pharyngitis, and bronchospasms. It can be acquired relatively easily without a medical prescription in various countries (Ecker et al., 2013). In human medicine, it is used for the treatment and prevention of respiratory, gastrointestinal, urinary, and skin bacterial infections. In animal medicine, amoxicillin is also used for the treatment and prevention of disease and to promote the growth of domestic (dogs, cats) and food animals (livestock, horses, pigs, goats, sheep, etc.) (Elizalde-Velázquez et al., 2016). The time it takes for the concentration of the amoxicillin in the plasma or the total amount in the body to be reduced by 50% is 1–1.5 h (terminal half-life of elimination), the excretion is renal and almost 80% of the unmetabolized amoxicillin is mainly excreted in the urine but is also secreted in milk. For these reasons, a wide range of concentrations, from traces to high levels, of this drug are found in surface water or wastewater.

Various analytical methods have been reported for the determination of amoxicillin such as HPLC (Pires de Abreu et al., 2003), spectrophotometric (Kishore et al., 2011), capillary electrophoresis (Hancu et al., 2016) as well as electrochemical methods (Fouladgar et al., 2011). The electrochemical sensor is a promising technology due to its simplicity and diversity. Moreover, the method has high repeatability, a good sensitivity, a low cost and is environmental friendly compared to the other analytical methods used for different types of analytes as drugs and food (Karimi-Maleh et al., 2016, 2020a,b; Karimi-Maleh and Arotiba, 2020).

In the present study, a mag-MIP with recognition sites for amoxicillin was synthesized, and this polymer was then used as a modifier in the carbon paste electrode for the detection of amoxicillin in milk and river water samples. Mag-MIP are materials based on magnetic nanoparticles (mag) coated with a molecularly imprinted polymer (MIP) that have attracted much attention due to their high magnetic character, chemical stability, ease of preparation, and low cost. The material has advantages such as a good sensibility, a high surface area, durability, and reusability as well as high selectivity toward amoxicillin.

## Materials and Methods

### Reagents and Solutions

All the reagents used in this work were of analytical grade. All solutions were prepared in ultrapure water (18 MΩ at 25°C) obtained from Milli-Q Direct-0.3 (Millipore). Ethyl acetate, triethyleneglycol (TREG), acetylacetonate [Fe(acac)_3_, 97%], ethanol absolute, ammonia solution (23-28% v/v), anhydrous toluene, tetraethyl orthosilicate (TEOS), and methanol (HPLC grade), sodium phosphate (monobasic and dibasic) purchased from Merck. Meanwhile, 3-methylpropyltrimethoxysilane (MPS), acrylamide (AAm), amoxicillin, potassium persulfate (KPS), *N, N*′-methylenebisacrylamide (MBAA), graphite, and mineral oil were acquired from Sigma–Aldrich. Ultrapure water (UP, 18.2 MΩ cm at 298 K) was used for all the experiments and was obtained from a Purelab Classic water purification system. The stock solution of amoxicillin (10 μmol L^−1^) was prepared in 0.1 mol L^−1^ sodium hydroxide solution.

### Mag-MIP and Mag-NIP Particles Synthesis

Magnetite nanoparticles were prepared following the polyol method (Cai and Wan, 2007; Vega-Chacón et al., 2016). Fe(acac)_3_ was dissolved in TREG using a mechanical stirrer in a three-necked round-bottomed flask equipped with a condenser and nitrogen gas which was passed through the reaction solution. The mixture was heated at 120°C for 30 min, then at 180°C for 30 min, and finally at 280°C for 60 min. The suspension was then cooled to room temperature.

A solution of ethanol and ethyl acetate (1:6) was added to the suspension. The nanoparticles were separated from the solution by magnetic-field-assisted sedimentation procedure employing a neodymium magnet and the supernatant was discarded. The nanoparticles were subsequently suspended in an ethanol-ethyl acetate solution. The magnetic separation and suspension processes were repeated until the supernatant was colorless. The nanoparticles were then dried at 50°C. The surface of the Fe_3_O_4_ nanoparticles was covered by silica through the Stöber sol-gel method (Stöber et al., 1968).

A quantity of 300 mg Fe_3_O_4_ nanoparticles was dispersed in 44 mL water-ethanol solution (1:10) using a sonicator. Then, 5 mL of ammonium hydroxide 28% (v/v) was added to the suspension, and immediately 2 mL of TEOS was added dropwise under continuous stirring and nitrogen gas flow. The reaction was allowed to proceed for 12 h at room temperature.

The obtained nanoparticles were separated by magnetic-field-assisted sedimentation, washed with water several times, and later were dried at 50°C. To introduce methacrylate groups on the surface, Fe_3_O_4_@SiO_2_ nanoparticles were functionalized with MPS. Fe_3_O_4_@SiO_2_ nanoparticles (250 mg) were sonically dispersed in 50 mL of dried toluene. Subsequently, 5 mL of MPS was added to the organic dispersion under continuous stirring and nitrogen gas flow. The reaction was allowed to proceed for 12 h at room temperature. The nanoparticles were then magnetically separated from the solution, washed with absolute ethanol several times, and dried at room temperature (Khan et al., 2018).

For the synthesis of mag-MIP, a mix of 1 mmol of amoxicillin (analyte) and 4 mmol of AAm (functional monomer) was dissolved in 80 mL of ultrapure water and the solution was stirred for 2 h. Promptly, a quantity of 300 mg of Fe_3_O_4_@SiO_2_-MPS was sonically dispersed and the suspension was stirred for 3 h under continuous stirring and nitrogen gas flow. Then, 100 mmol of MBAA (crosslinker) was added to the suspension and nitrogen gas was bubbled for 10 min. Afterward, 0.185 mmol of KPS (radical initiator) was added to the suspension and the reaction was allowed to progress for 3 h at 60°C. The mag-MIP was separated by vacuum filtration, washed many times with absolute ethanol, and dried at room temperature. The amoxicillin templates were removed using an extraction system (Soxhlet) with solutions of methanol/water (90:10) and methanol/water (70:30), v/v for a period of 8 h. The washing was repeated until no presence of analyte was verified by HPLC during the washing process.

**Scheme 1:**
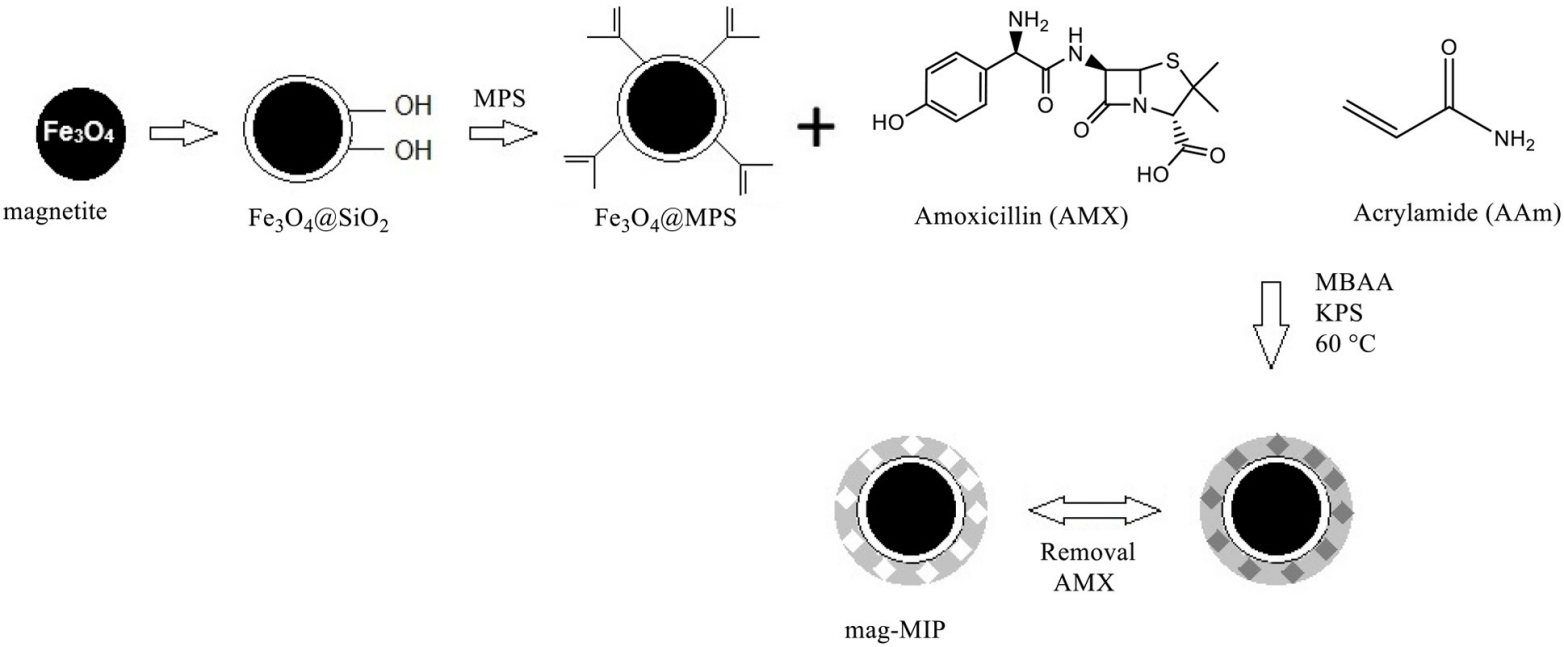
Representation of the steps followed for the synthesis of mag-MIP.

The mag-NIP was prepared in the same way but without the addition of amoxicillin. A schematic representation of all the steps is provided in Scheme 1.

### Characterization of Mag-MIP and Mag-NIP

The scanning electron microscopy (SEM) model: (JSM-7500F microscope) was used to characterize the surface morphology of synthesized nanomaterials.

A high-resolution Transmission electron microscope (Model: CM200 super twin with a resolution of 1.9A. Company: Philips) was used to evaluate the mag-MIP and mag-NIP particles.

To elucidate the crystalline phase we conducted X-ray diffractometry (XRD) using a Bruker D2 Phaser using Cu Kα (λ = 1.5418 Å) radiation source with a scanning window of 20–90° at 0.4°/s. FTIR spectra were obtained using a Shimadzu FTIR-8400S Prestige-21 spectrophotometer, with the sample in a matrix of KBr to show the functionalization and polymerization through the presence of the different functional groups. Magnetic properties were analyzed by measurements of vibrating sample magnetometry (VSM). The equipment was built for research (Jordán et al., 2018). It shows a hysteresis plot for a magnetic field up to 2,500 Oe with a resolution of ~2 × 10^−4^ emu.

### Preparation of Mag-MIP/CPE Sensor

The Mag-MIP/CPE sensor was prepared as described in previous works (Wong et al., 2015; Pizan-Aquino et al., 2020) using a mixture of 85 mg of graphite, 15 mg of mag-MIP (or mag-NIP), and 1.0 mL of deionized water. The mixture was carefully homogenized for 20 min, employing a mortar and pestle, and kept for 12 h at room temperature to become dehydrated. Afterward, 80 μL of mineral oil was added dropwise, to obtain each modified paste (Khan et al., 2019).

The prepared working paste was used to fill the empty hole of the Teflon® working electrode (WE) where a Pt disk was used to provide the electrical connection. The prepared modified electrodes were nominated as mag-MIP/CPE, mag-NIP/CPE, and CPE (carbon paste electrode), prepared for comparison reasons.

### Optimizing the Mag-MIP/CPE Sensor

Electrochemical characterization of amoxicillin was carried out using square wave voltammetry (SWV), in a potentiostat (Model μAutolab Type III, Autolab/Eco Chemie) and with a three-electrode system: mag-MIP/CPE sensor as the working electrode (WE), a commercial Ag/AgCl (KCl_sat_) as the reference electrode (RE, Analion®) and a platinum wire as the counter electrode (CE).

The effect of potential step increments was studied over the range from 1 to 10 mV, the amplitude was varied over a range from 25 to 100 mV and the frequency was modulated between 8 and 30 Hz with a fixed scan rate of 10 mV s^−1^. Additionally, to explore the mechanism of amoxicillin oxidation on the mag-MIP/CPE sensor, some SWV experiments were made at different pH values ranging from 5.0 to 9.0 in a phosphate buffer solution with a concentration of 1.0 mmol L^−1^ to obtain the dependence of the electrochemical oxidation of amoxicillin on pH.

### Application of the Mag-MIP/CPE Sensor to Real Samples

The performance of the mag-MIP/CPE sensor was evaluated by testing river water and milk samples. To this aim, some river water samples were collected from the Gregorio river located in the municipality of São Carlos at the São Paulo State in Brazil. No pre-treatment processes were applied in the samples. The samples were placed in 5 mL capacity flasks and spiked with a standard solution of amoxicillin at three concentration levels. For this, 400 μL of each sample were diluted in 0.1 mol L^−1^ phosphate buffer solution (pH 7.0) and analyzed in the electrochemical system with a capacity of 10 mL.

The milk sample was purchased from a supermarket in the same city. The samples were spiked with a standard solution of amoxicillin at 2.5 and 57 μmol L^−1^ concentration level and analyzed in the electrochemical system (at concentrations corresponding in the cell of 3.0 × 10^−6^ and 3.0 × 10^−5^ mol L^−1^). The samples were submitted to the centrifugation process and the supernatant was used in the electrochemical tests.

## Result and Discussion

### Characterization of the Mag-MIP

**Figure 1:**
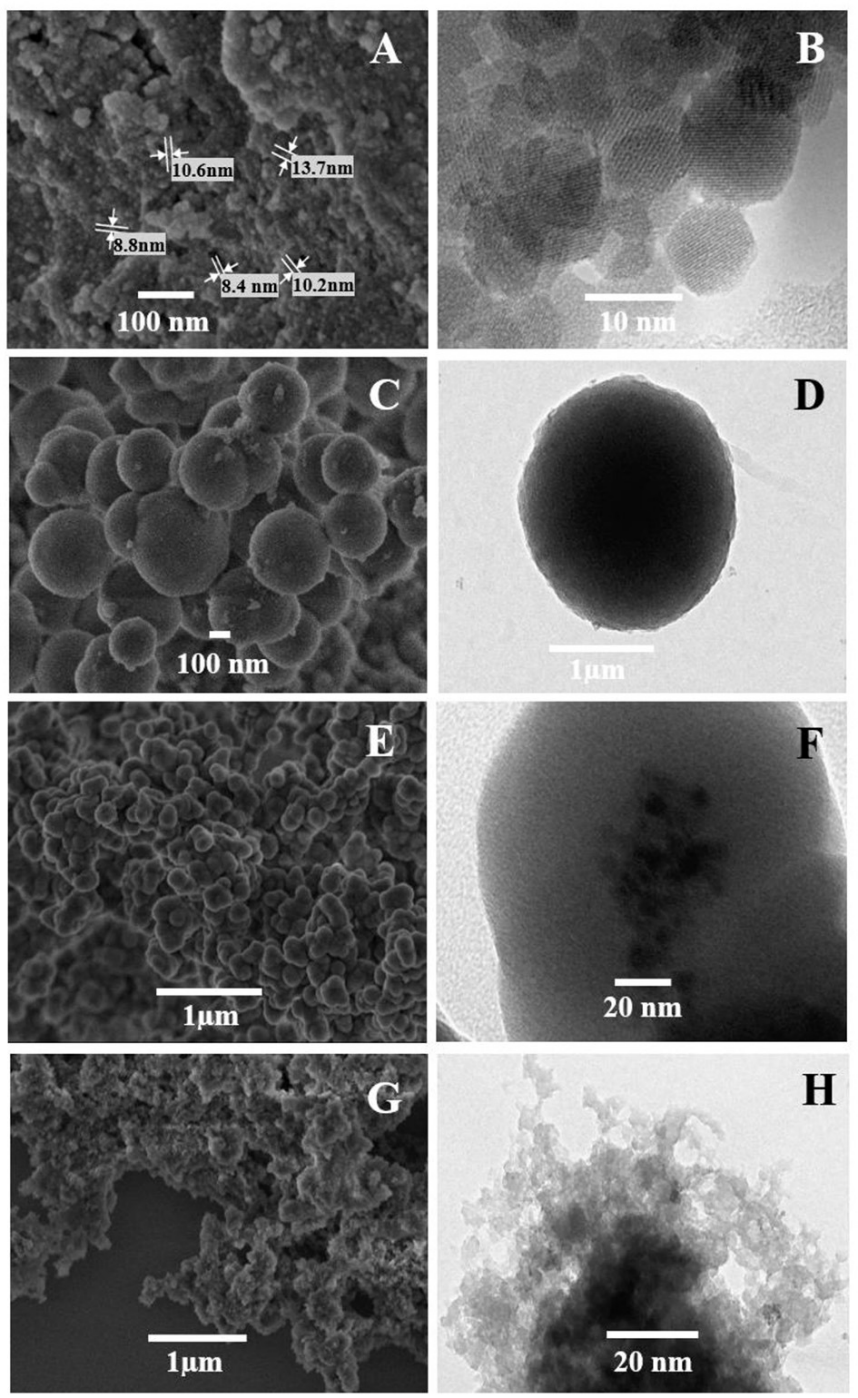
SEM **(A,C,E,G)** and TEM **(B,D,F,H)** micrograph of the materials: **(A,B)** Fe_3_O_4_, **(C,D)** Fe_3_O_4_@SiO_2_, **(E,F)** Fe_3_O_4_@MPS, and **(G,H)** mag-MIP.

The SEM and TEM images of the mag-MIP are shown in Figure 1, where the morphology and particle size of materials in different stages of synthesis. SEM and TEM micrographs showed the presence of magnetite nanoparticles (Fe_3_O_4_) with an average diameter of 10 nm and apparently with a monodisperse distribution (Figures 1A,B). In this work, magnetite was successfully synthesized by the polyol method which replaces the traditional and commonly used co-precipitation method for the preparation of superparamagnetic water-soluble nanoparticles, used in various biomedical applications (Cai and Wan, 2007; Vega-Chacón et al., 2016). The SEM and TEM images of silica-coated magnetite nanoparticles are presented in Figures 1C,D, respectively. The images indicate that the iron oxide core was coated for an inert silica shell taking a total spherical shape with size up to 300 nm, however, the coated thickness could be varied by changing the amount of TEOS (Laurent et al., 2008), modifying the concentration of ammonium and the ratio of tetraethoxysilane (TEOS) to H_2_O. The silica shell stabilizes the magnetic nanoparticles and prevents their aggregation in a liquid, as well as improves their chemical stability and provides better protection against toxicity (Lu et al., 2007). An additional covering with MPS is presented in Figures 1E,F, which show the spherical-like particles of Fe_3_O_4_@SiO_2_-MPS in which no considerable change in particle size was observed. Here, silanol groups (SiO_2_) are functionalized by silanes that contain the multiple bonds indispensable for the further polymerization process (Ansari, 2017). Finally, Figures 1G,H show the polymerization of the MIP on the agglomerated magnetite nanoparticles (mag-MIP), forming a polymeric layer and full coverage of the magnetic core.

**Figure 2:**
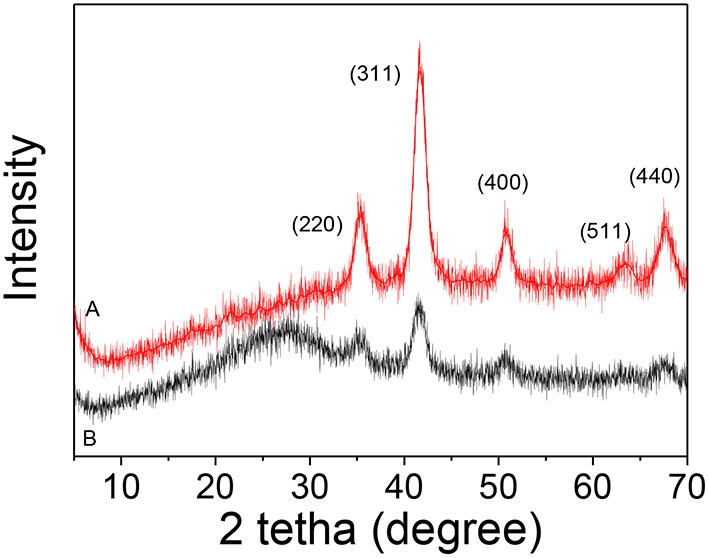
XRD patterns: **(A)** Fe_3_O_4_ and **(B)** Fe_3_O_4_@SiO_2_.

Figure 2A shows the XRD pattern of iron oxide nanoparticles synthesized by the polyol method, which observed the highly crystalline cubic spinel structure (JCPDS 19-0629) assigned to magnetite and the high purity of the magnetic nanoparticles (Fe_3_O_4_) since no other signal was detected. The silica-coated magnetite (Fe_3_O_4_@SiO_2_) shows in its diffraction pattern (Figure 2B) an amorphous hump corresponding to the SiO_2_ around 25° (Gao et al., 2011) and the rest of the peaks remaining unchanged, from which is inferred that the coating with silica did not affect the starting crystalline structure of the Fe_3_O_4_ (Chang et al., 2012).

**Figure 3:**
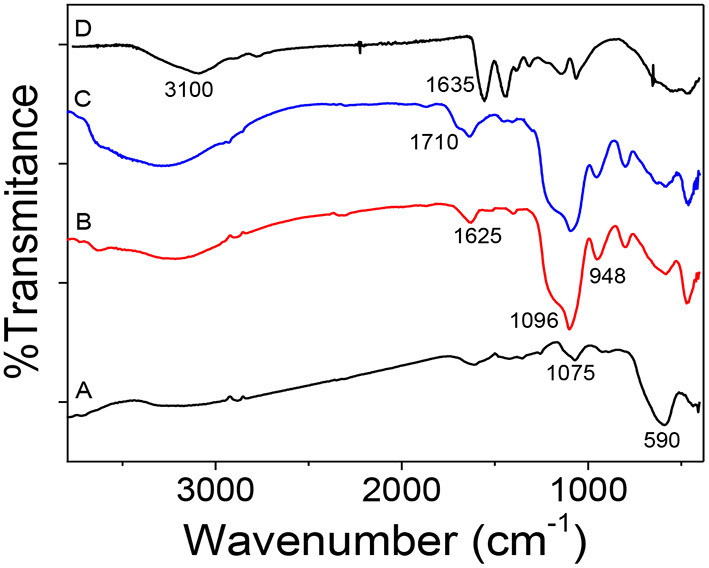
FTIR spectra: **(A)** Fe_3_O_4_, **(B)** Fe_3_O_4_@SiO_2_, **(C)** Fe_3_O_4_@MPS, and **(D)** mag-MIP.

Figure 3 shows the FTIR spectra of the evolution from Fe_3_O_4_ to mag-MIP to verify the functional groups that appear during the synthesis. In Figure 3A, the inverse spinel structure of magnetite was again confirmed by IR bands, from the vibrations Fe–O–Fe at 590 and 1,075 cm^−1^ characteristic of vibration of Fe-O stretching at tetrahedral sites. In Figure 3B, for magnetite coated with SiO_2_, some characteristic peaks were identified at 1,096 cm^−1^, a sharp and intense peak, due to the asymmetric vibration of Si–O–Si and at 948 cm^−1^, attributed to the Si-O-H stretching vibrations. A small peak at 1,625 cm^−1^ appears to be attributed to the hydroxyl group revealing the formation of silicon shell (Chang et al., 2012). For the functionalized (silanized) sample, the presence of a shoulder observed at 1,710 cm^−1^ could be assigned to the carbonyl group C=O from the MPS (Figure 3C). Finally, a wide, broad band was observed between 3,100 and 3,500 cm^−1^ corresponding to the NH bonds of a primary amide of the polyacrylamide formed, and the peak at 1,635 cm^−1^ would correspond to the vibration of the C=O. The weak peaks at 1,120 and 1,213 cm^−1^ correspond to C-N vibration. These results confirmed that the coating with acrylamide-based polymer was performed satisfactorily (Figure 3D).

**Figure 4:**
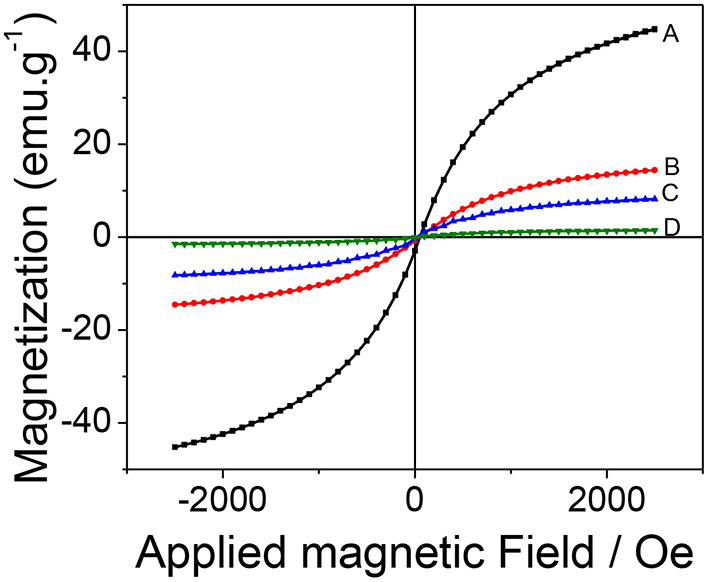
Magnetization hysteresis curves: **(A)** Fe_3_O_4_, **(B)** Fe_3_O_4_@SiO_2_, **(C)** Fe_3_O_4_@MPS, and **(D)** mag-MIP.

The magnetic behavior of the synthesized material was studied by magnetization curves (VSM) at 300 K. Figure 4 shows that all the curves present the same characteristics: the absence of hysteresis and coercivity, suggesting that all the samples are superparamagnetic. The saturation magnetization (Ms) value obtained for magnetite was 44.5 emu g^−1^ which drastically decreased to 14.3, 8.3, and 1.3 emu g^−1^ for Fe_3_O_4_@SiO_2_, Fe_3_O_4_@SiO_2_-MPS, mag-MIP, respectively. This decrease in magnetization values can be attributed to the increase of the coating layers of non-magnetic materials on the magnetite and therefore, the size, but keeping the superparamagnetic nature (Pan et al., 2014; Uzuriaga-Sánchez et al., 2016).

### Development of the Electrochemical Sensor Modified With Mag-MIP

Square wave voltammetry (SWV) technique was used to detect amoxicillin in a phosphate buffer solution of pH 7.0 using a mag-MIP/CPE sensor under optimized experimental conditions. The best experimental values for the SWV parameters were: step = 5 mV, amplitude = 50 mV and frequency= 15 Hz.

**Figure 5:**
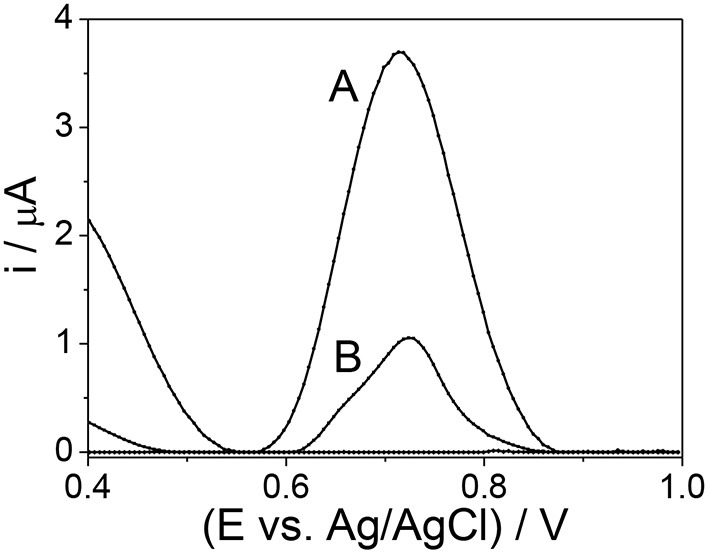
SWM voltammograms **(A)** mag-MIP/CPE and **(B)** mag-NIP/CPE in the presence of 10 μmol L^−1^ amoxicillin in phosphate buffer solution at pH 7.0.

The response profile by SWV of mag-MIP/CPE and mag-NIP/CPE in the presence of 1.0 mmol L^−1^ of amoxicillin at 0.72 V vs. Ag/AgCl is shown in Figure 5. The remarkable difference between mag-MIP and mag-NIP sensors is revealed and could be attributed to the presence of the selective cavities in the mag-MIP which increased the sensitivity in the amoxicillin detection, this behavior is also outlined in literature by other authors (Montoya et al., 2015; Yang et al., 2020). The expected profile of the non-selective polymer (mag-NIP/CPE) and CPE (electrode carbon paste) was not satisfactory as compared to magnetic selective polymer (mag-MIP/CPE). This fact can be explained by the lack of selective pore toward the analyte on the surface of the mag-NIP/CPE and in the CPE.

#### Effect of pH

**Scheme 2:**
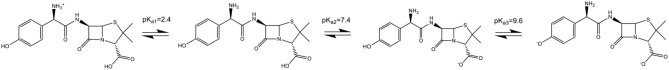
Protonated and deprotonated forms of amoxicillin.

The pH is one of the most significant factors that could affect the performance of the electrochemical process. In the amoxicillin molecule (Scheme 2) when pH < pK_a1_ (2.4), the molecule accepts a proton forming an ion with a positive charge, causing electrostatic repulsion on the electrode surface. In the situation where the pK_a1_ (2.4) < pH < pK_a2_ (7.4), the molecule is not charged or ionized. When pK_a2_ (7.4) < pH < pK_a3_ (9.6), the molecule losses a proton forming an ion with a negative charge. When pH > pK_a3_ (9.6), the amoxicillin losses two protons forming an ion with two negative charges, forming a phenolic anion that cannot be oxidized (Zhao et al., 2019).

**Figure 6:**
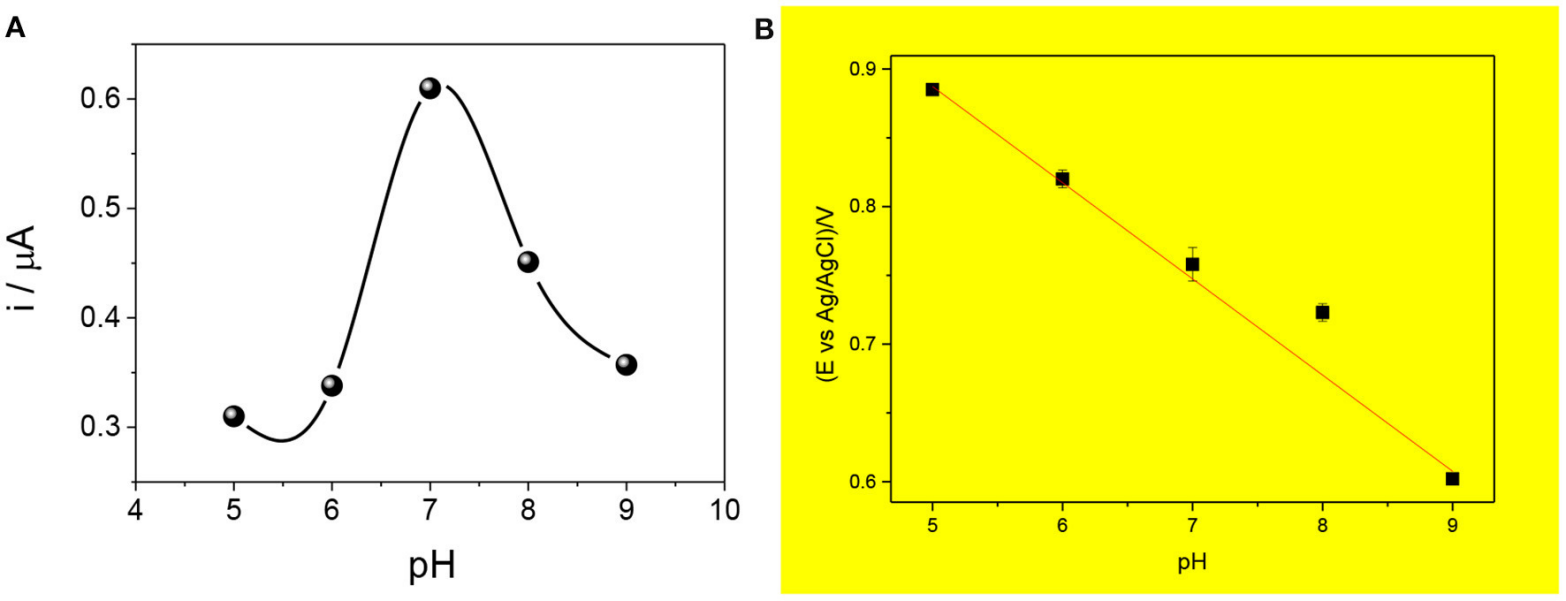
Effect of pH on the current intensity **(A)** and the anodic potential peak **(B)** for the proposed mag-MIP/CPE sensor. Measures carried out using SWV and 2.5 μmol L^−1^ amoxicillin at different pH (5, 6, 7, 8, and 9).

The effect of pH on the response of the sensor was evaluated, as shown in Figure 6. As can be observed in Figure 6A, the pH increases up to 7.0 in which the signal is the highest, so the adsorption is highly favored. Then, the current decreases at pH near 8.0 and 9.0 when the amoxicillin is deprotonated and the phenolic group oxidization diminishes.

Figure 6B shows the influence of pH in the peak potential value, revealing a linear relationship with a slope of −0.067 V pH^−1^ that is value next to the theoretical Nernstian slope of −0.059 V pH^−1^. This indicates that the redox process in this sensor corresponds to a system where the same number of protons and electrons are involved (Zeinali et al., 2018; Wong et al., 2020).

**Scheme 3:**
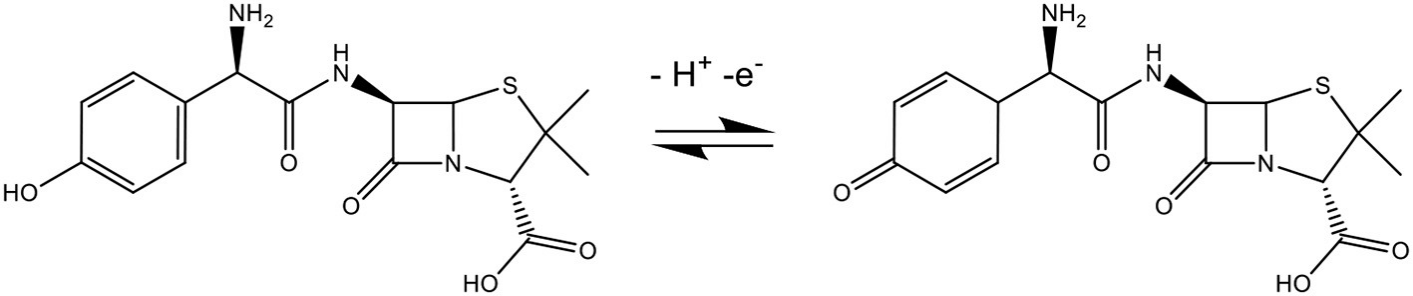
Electrochemical oxidation mechanism of amoxicillin.

The electrochemical mechanism is shown in Scheme 3, where the oxidation of the phenolic group present in the amoxicillin is adsorbed on the electrode surface.

#### Analytical Curve for Amoxicillin

**Figure 7:**
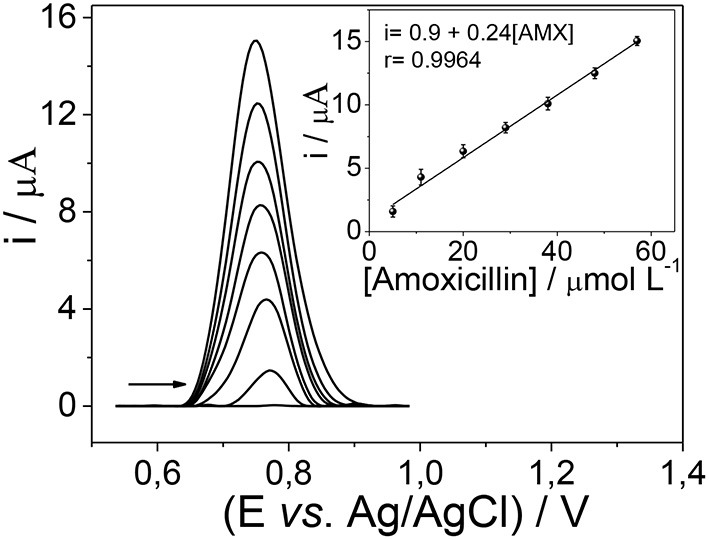
Electrochemical determination by SWV of amoxicillin using the mag-MIP/CPE sensor.

To quantify amoxicillin in different matrices, the electrochemical analysis was made by the square wave voltammetry technique. Figure 7 depicts a change in the magnitude of anodic peak current as a result of the addition of different amoxicillin concentrations. Under optimized conditions, the sensor had a response linear range from 2.5 to 57 μmol L^−1^ amoxicillin (*r*^2^ = 0.996), with a limit of detection and limit of quantification of 0.75 and 2.48 μmol L^−1^, respectively.

#### Repeatability and Selectivity Studies

The repeatability was tested by 10 electrochemical measurements by SWV in presence of 10 μmol L^−1^ of amoxicillin and was evaluated in terms of the relative standard deviation (RSD, Equation 1). The value obtained in this analysis was lower than 4%, indicating good repeatability and stability in the determination of this antibiotic using the proposed sensor.

(1) $$\begin{array}{l} \text{RSD}=\;\frac{\text{S}}{\bar{\text{X}}}\text{ x }100 \end{array}$$

In Equation (1), *S* represents the standard deviation and $\bar{\text{X}}$ represents the average value.

The selectivity of the sensor was tested by comparing the SWV signal of the analyte against other chemicals heavily consumed in daily life (urea, caffeine, uric acid, ciprofloxacin, ascorbic acid, and sodium chloride). These studies were carried out in binary mixtures (analyte and other compounds) under optimized conditions in the concentration of 5 μmol L^−1^. The results showed no interference in the electrochemical signal of amoxicillin for triplicate experiments since in the presence of interfering compounds obtained error values lower than 5% to the sensor signal.

#### Application of the Sensor in Skimmed Milk and River Water Samples

The mag-MIP/CPE sensor was applied to quantify amoxicillin in skimmed milk and river water samples, prepared as described in the experimental section. The analysis was performed in triplicate experiments and the number of amoxicillin present in the samples was calculated using the calibration curve. Table 1 shows the recovery values obtained in the quantification of amoxicillin in these samples. The recovery values obtained were close to 100% using the SWV technique, showing good applicability of the proposed sensor in these matrices.

**Table 1:** Recovery rates obtained from the use of the proposed mag-MIP/CPE sensor in the analysis of regional river water and skimmed milk samples spiked with known amoxicillin concentrations.

**Samples**	**Place obtained**	**Added/mol L^**−1**^**	Proposed method^*^/mol L^**−1**^	**Recovery (%)**
River water	Gregório (São Paulo state—Brazil)	3.0 × 10^−6^	(3.0 ± 0.3) × 10^−6^	100.3
		8.0 × 10^−6^	7.98 × 10^−6^	98.8
		3.0 × 10^−5^	(3.0 ± 0.2) × 10^−5^	100.2
Skimmed milk	Local market (São Paulo state–Brazil)	3.0 × 10^−6^	(2.9 ± 0.2) × 10^−6^	96.7
		8.0 × 10^−6^	7.9 × 10^−6^	97.5
		3.0 × 10^−5^	(2.9 ± 0.3) × 10^−5^	96.7

* *Standard deviation values corresponding to n = 3*.

## Conclusion

The present work has reported on a simple and low-cost sensor for the quantification of amoxicillin in skimmed milk and river water samples. The use of mag-MIP contributed to the increase of the electrochemical signal from amoxicillin when compared with its non-impressed counterpart mag-NIP, as well as when the mag-MIP has a high surface area as compared with mag-NIP. The various factors influencing electrochemical determination such as pH, dose, contact time, and the concentration of an analyte, were optimized to get a maximum anodic current peak. The use of magnetic particles combined with MIP boosts its surface area which helps peak current increase. It is also easy to wash this material by using an external magnet instead of filtration.

The proposed sensor exhibited high stability, fast analysis time, good selectivity, and sensibility. Finally, the main advantages of the proposed method were the ability to renew the electrode surface by simple polishing allowing further analysis.

## Data Availability Statement

The raw data supporting the conclusions of this article will be made available by the authors, without undue reservation.

## Author Contributions

RL was responsible for experimental studies. SK and AW were responsible for experiments, revision of experiments, and initial redaction of the paper. MS was responsible for the submission, final revision, and financial support to this research. GP was responsible of the correction of the final version and for the financial support. All authors contributed to the article and approved the submitted version.

## Conflict of Interest

The authors declare that the research was conducted in the absence of any commercial or financial relationships that could be construed as a potential conflict of interest.
